# Voyages in Vacation. I

**Published:** 1888-09-29

**Authors:** 


### Voyages in Vacation.—I.

By One in Search of Restful Restoratives.

Holidays are necessary, and being necessary are regarded by many of those who take them year by year as growing evils, in proportion to the age attained by them. Indeed, life presents few greater paradoxes than that of the man whose desire to leave his work decreases in inverse proportion to the pressure under which he labours, and the consequent necessity for him to altogether give up all business engagements for a time. The greater the responsibility of the individual, be he premier, statesman, financier, or professional man, the more will those whom he works with or for cling to every chance of consulting him by wire or letter in his absence if an emergency arises, so long as he is anywhere within reach. Responsibility is a rich dish, and those who have a plateful may thankfully ladle it out to as many as wish to share or taste its grinding burden. So, when voluntarily or by compulsion, *i.e.*, by doctor's orders, the overworked and weary man is at last brought to leave his work for a vacation, he should strive to find some place of retirement where he will not be bothered by telegrams, nor worried by queries from clients or subordinates, which might well be held over till his return. Few methods are so admirably suitable to men in the position we are considering as a sea voyage. Many have dwelt upon the horrors of sea sickness, and the monotony of life at sea, but few have brought out the real advantages and enjoyments of a voyage in a good ship, under proper auspices and conditions; yet the enjoyments and amusements of sea travelling are neither few nor by any means to be despised.

Since Mr. Gladstone was feted at sea by Sir Donald Currie, and so cured of a troublesome hoarseness, some of the multitude have turned their thoughts seaward, and shipowners have recognised this change of feeling on the part of the public by providing excellent means of transit and many fresh scenes of travel. An old P. and O. steamer was purchased by an enterprising gentleman (Captain Leview), and chartered some years ago as the steam yacht *Ceylon*, in which excursions have been made by hundreds of leisured and well-to-do people to north, east, and south. Encouraged by the welcome extended to this enterprise, a second steam yacht, the *Victoria*, a new vessel of about 2,000 tons register, superbly furnished, and well found in every way, was commissioned entirely for pleasure traffic by Mr. Jones and Captain Lunham, and has proved a conspicuous success. Carrying seventy passengers when full, the *Victoria* combines many of the advantages of a private yacht with the comforts and nearly the ample space of a P. and O. steamer. All these advantages are procurable at a cost which is, on the whole, so reasonable as to be surprising. The Cunard and other lines have likewise organised excursions to various places at an inclusive and moderate cost. The changes thus introduced for the benefit and comfort of travellers who desire to make their holidays of real assistance to them in the discharge of their subsequent every-day life are too recent to be fully recognised by the many, but, as usually happens, the more wide awake and enterprising, especially some of those wealthy residents in northern cities who are deservedly famed for their astuteness, have not been slow to take passage in these pleasure yachts to their own satisfaction and advantage.

The insular prejudice of the British public causes it to believe that, as the rain has fallen immoderately in England during the summer and autumn, whilst the weather has in other respects been most unpropitious, all prudent holiday-seekers will take this as the index of weather elsewhere, and will so stay at home and save their money for a better season. As a matter of fact, however, probably few seasons abroad have had better weather on the whole than that of the latter half of August and September, 1888. With a temperature not too hot to be unbearable, nor too cold to render it impossible to sit in the open air with comfort, the traveller by sea has been pleasantly surprised to find how enjoyable and healthful a pursuit yachting really can be to the weary and overworked, as well as to the idle. Those who have had the good fortune to yacht in the North and Baltic Seas during the past two months will readily admit that the weather has been perfect, and the result to their personal health remarkable. From Tilbury to Copenhagen, about three days' steam, gives the passenger a restful and pleasant experience of the sea. He will be astonished at the extent of the Baltic trade, as evidenced by the very numerous steamers and vessels almost continously encountered on the voyage. These ships are of all types, owned for the most part by Englishmen, but there is abundant evidence that other nations are competing with us to an ever-increasing extent to secure as large a share of the carrying trade of the world as possible. Danish and German vessels are, of course, the most numerous of the foreigners met with. Off the Dogger Bank in the North Sea a strange and novel sight meets the eye of the voyager. This consists of hundreds of boats with all sails set, which sail in line in opposite directions and at full speed, as they fish for the cod, which are found in inexhaustible quantities off this Bank. Subject to no immediate control or authority, except that of the captain of the fleet elected by themselves, these hardy North Sea fishermen successfully ply their trade for months at a time, under difficulties and dangers few realise and none would by choice elect to face. No prettier or more effective sea picture can be imagined than this fleet of fishing-boats off the Dogger Bank, and it is well worthy of the attention of the first sea painter of the day, for whose brush it would supply with admirable material. Our readers will be acquainted the name, at any rate, of the society which undertakes to provide Missions to the Deep Sea Fishermen. It is advertised more extensively than any other society of the kind, and performs, it is to be hoped, some useful purpose to the fishermen, for it states that hospital ships and more than one mission vessel are provided by it for their use and benefit. In these circumstances it is not a little remarkable that the work of this society off the Dogger Bank seems to be so unobtrusively performed that neither mission-boats nor hospital-ships appear to carry any distinctive flag or sign by which they can be distinguished from the ordinary fishing-boats. At least, those who have seen the fleet off the Dogger Bank on a clear day and within easy distance, as the writer did on the 1st of September, will probably not have been able to make out one mission or hospital ship, although the steamer which accompanies the fishing fleet was plainly distinguishable in the midst of the fleet. As the steamer approaches Copenhagen, numerous ships of war were encountered, including German, Danish, and Russian ironclads, and possibly a royal or imperial yacht also. There are few prettier sights on a bright fine day than the harbour of Copenhagen with its approaches, and probably the only place where vessels would appear more numerous and picturesque than there is in Boston Harbour, U.S.A., when the wind is blowing shorewards, and some hundreds of sail of all kinds are waiting, with sails set, for that slight change in the wind which will enable them to set out for the ports which they desire to reach.

*(To be continued.)*

